# Chemical Composition and Nutritive Benefits of Chicory* (Cichorium intybus)* as an Ideal Complementary and/or Alternative Livestock Feed Supplement

**DOI:** 10.1155/2017/7343928

**Published:** 2017-12-13

**Authors:** Ifeoma Chinyelu Nwafor, Karabo Shale, Matthew Chilaka Achilonu

**Affiliations:** ^1^Department of Agriculture, Faculty of Health and Environmental Sciences, Central University of Technology, Free State, Private Bag X20539, Bloemfontein 9300, South Africa; ^2^Department of Environmental Sciences, Faculty of Natural Sciences, Mangosuthu University of Technology, P.O. Box 12363, Jacobs, Durban, Umlazi, KwaZulu-Natal 4026, South Africa

## Abstract

Chicory is a perennial plant grown in different parts of the world, used as forage for livestock, as folklore remedies, or as a vegetable addition in human diets. There are several varieties of the chicory plant, known differently globally due to its numerous medicinal, culinary, and nutritional qualities. Most parts of the plant contain a potpourri of nutrients ranging within carbohydrates, proteins, vitamins, minerals, soluble fiber, trace elements, and bioactive phenolic compounds, which are responsible for the various nutritive, prophylactic, and therapeutic qualities of chicory. Inulin, coumarins, tannins, monomeric flavonoids, and sesquiterpene lactones are some of the major phytocompounds mostly found in chicory plants. The health-promoting activities attributed to chicory comprise, among others, anti-inflammatory, anticarcinogenic, antiviral, antibacterial, antimutagenic, antifungal, anthelmintic, immune-stimulating, and antihepatotoxic and its antioxidative qualities. As a versatile plant, chicory's chemical composition and use as a suitable livestock feed supplement or as an alternative feed ingredient (AFI) are thus reviewed.

## 1. Introduction

**Figure 1:**
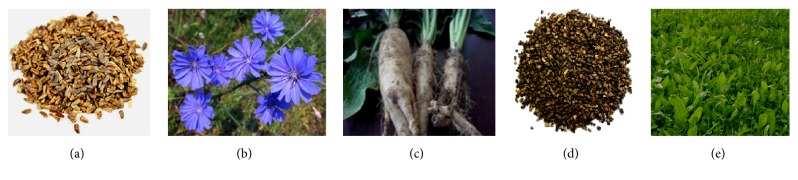
Useable parts of the chicory plant. (a) Seeds, (b) flower, (c) roots, (d) roasted roots, and (e) chicory field [28].

The use of cereals in animal feeds competes with human nutrition, and some of the cereals are too expensive. Therefore, the introduction of alternative feedstuffs that could overcome the challenges of production cost in the livestock industry as well as impact positively animal health, production yield, and product quality has become necessary. Some of these AFI such as chicory parts (seeds, leaves, and roots) (Figure 1) have successfully been used as supplements in small ruminant diets, without compromising animal performance.* Cichorium intybus*, a perennial herbaceous plant of the Asteraceae family, has been used for ages as livestock forage in various parts of the world. Popularity of chicory is steadily growing owing to its numerous medicinal, culinary, and nutritional qualities.

The leaves and flowers are usually used as vegetables in salads and the roots are used as a coffee substitute, livestock feedstuff, or pet food [1]. Chicory extracts are sometimes added to alcoholic and nonalcoholic beverages to improve taste [2], while the inulin rich tuberous roots can be converted to alcohol [3]. In some countries, parts of the chicory plant are used as ethnoveterinary remedy for bodily ailments and disorders and for prophylactic purposes in both humans and livestock [4, 5]. Since fresh chicory roots have a rather bitter taste, the roots are normally debittered by boiling, drying, baking or roasting, and soaking in water or citric acid solution and then chopped or milled before being used as coffee blends, feed, or a functional food ingredient [1, 6–8].

Chicory root contains some phytochemicals such as inulin (starch-like polysaccharide), coumarins, flavonoids, sesquiterpene lactones (lactucin and lactucopicrin), tannins, alkaloids, vitamins, minerals, and volatile oils [9]. The secondary metabolites (flavonoids, tannins, and coumarins) found in chicory have been reported to demonstrate some biological activities such as antioxidant, anticancer, anti-inflammatory, antiparasitic, antihepatotoxic, which impact positive health effect on humans and livestock [10, 11]. Inulin is a polymer of fructose with *β*-(2-1)-glycosidic-linkage [12], which accounts for up to 68% of the total compounds present in fresh chicory roots [13, 14]. As a prebiotic, inulin is low in calorie and dietary fiber, making it a good replacement for sugar and an ideal component for diabetic nutrition [15, 16].

Furthermore, animal performance on forage chicory is similar to that of legumes and superior to grass‐based pastures [11]. According to [17], chicory increases milk production when offered as a supplement to pasture. As reported elsewhere [18–21], grazing chicory is known to decrease some internal parasites in livestock and therefore has potential to reduce the use of anthelmintics. This paper reviews and summarizes research opinions on chicory's chemical constituents and associated nutritional properties, which makes it an ideal complementary and/or alternative livestock feed supplement.

## 2. Phytochemical Constituents of Chicory

A recent reduction in the incidences of chronic diseases is largely associated with high dietary ingestions of varied vegetables and fruits [22]. The health-promoting factors in these plant parts, commonly called phytochemicals, have extensive biological activities such as antioxidant, anticancer, anti-inflammatory, and *α*-glucosidase inhibition. The very common phytocompounds are phenolic acids, which include chlorogenic acids, and flavonoids (anthocyanins, flavonols, flavanone, and flavan-3-ols). The plant polyphenols usually occur as glycosides, which makes them less reactive and easier to store in the cell vacuole [23]. Cleavage of the glycosidic-linkage and associated rearrangement reactions releases the following sugar residues: hexose, glucose or galactose, deoxyhexose, rhamnose, pentose, xylose or arabinose, and glucuronic acid [23]. The anthocyanins have been reported to reduce the risk of coronary heart disease in animals by exhibiting arterial protection, inhibition of platelet aggregation, and protection of endothelial tissues [23–26].

**Figure 2:**
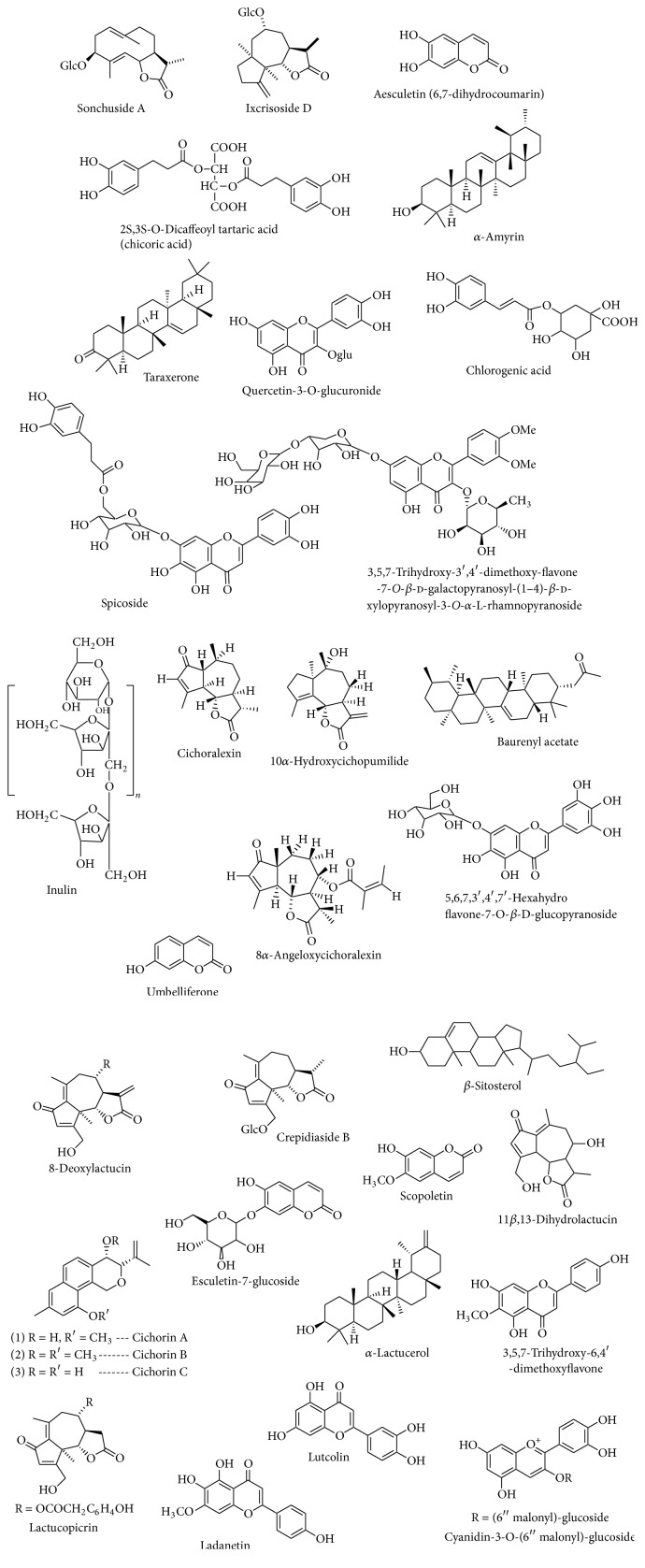
Identified phytoconstituents of chicory [11].

Many studies have shown that chicory is an embodiment of animal health-promoting phytocompounds, which includes condensed tannins. However, when the tannins are present in high concentrations in the diet, it can negatively affect animal productivity [27]. Another study by Carazzone et al. (2013) [23] recorded the extraction of phenolic acids and flavonoids from several types of* Cichorium intybus *var.* silvestre* and the characterization of the compounds using high-performance liquid chromatography-electrospray ionisation/mass spectrometry. Sixty-four compounds were detected, which include several hydroxycinnamic acid derivatives comprising eight mono- and dicaffeoylquinic acids, three tartaric acid derivatives, thirty-one flavonol and two flavone glycosides, and ten anthocyanins as well as several isomers of caffeic acid derivatives. The recent review report of [11] focused on ethnomedicinal, botanical, and phytopharmacological aspects of* Cichorium intybus* and identification of some phytoconstituents of chicory as presented in Figure 2.

Massoud et al. (2009) [1] investigated the chemical composition of the leaves and roots of* Cichorium Intybu*s and the representative phytoconstituents presented as macronutrients, micronutrients (essential minerals), and phenolic compounds in Tables 1, 2, and 3, respectively.

Many researchers noted that fresh chicory roots contain, by dry weight, 68% of inulin (a polysaccharide similar to starch), 14% sucrose, 5% cellulose, 6% protein, 4% ash, and 3% other compounds [13, 30–32]. These researchers also noted that dry chicory root extract contains, by weight, approximately 98% inulin and 2% of other compounds. A study by Soobo (2005) [33], also agreed that chicory root is high in inulin-type fructan and oligofructose. Chemically, inulin is a polydisperse-(2,1)-fructan and can be converted to fructose and glucose by hydrolysis. Chicory contains affluent quantities of fructose (up to 94%), which is a long chain carbohydrate, consisting of 22–60 fructose molecules with a terminal glucose molecule [34]. High quantities of sesquiterpene lactones, such as lactucin, 8-deoxylactucin, lactucopicrin, and 11ß-dihydro-derivatives, are hugely responsible for their bitterness [35]. This was confirmed by some researchers [36], who isolated sesquiterpene lactones, a (+)-germacrene from chicory roots. According to a study by [36], roasted chicory roots contain various compounds like 2-acetylpyrrole, furfural, phenyl acetaldehyde, phenyl acetic acid, vanillin, pyrazines, benzothiazoles, aldehydes, aromatic hydrocarbons, furans, phenols, organic acids, and small quantities of insole alkaloid (ß-carbolines), harmane and norharmane. Chicory root extract, produced by removing the insoluble fraction of milled dry root in water by filtration and centrifugation, contains volatile oils, fatty acids, alkaloids, triterpenes, flavonoids, latex, tannins, and saponins [37]. Three new benzo-isochromenes (Cichorins A, B, and C) were isolated from chicory roots [38, 39]. According to these researchers, the roots also contain tannins, fatty acids (mostly palmitic and linoleic), pectin, *α*-lactucerol (taraxasterol), cichoriin (esculetin-7-glucoside), sugars (especially fructose and mannose), fixed oils, choline, and others.

Chicory seeds contain a rich potpourri of nutrients ideal for both ruminant and monogastric nutrition. Ying and Gui (2012) [40] stated that most varieties of chicory seeds have high amounts of crude protein which is more than 19% of the dry weight and 1.6–2.4 times higher than the value in most conventional grains, like wheat, rice, corn, and barley. These authors also noted that good sources of most essential amino acids like methionine, lysine, leucine, isoleucine, phenylalanine, and so on, which are recommended for an ideal dietary protein, can be obtained from chicory seeds. Moreover, the seeds contain abundant demulcent oils, a good source of both saturated and unsaturated fatty acids [41], having the essential linoleic acid (18:2n-6) content of more than 76% of the total fatty acids profile which includes the monounsaturated oleic acid (18:1n-9), stearic acid (18:0), and palmitic acid (16:0) [40]. Relatively high levels of essential minerals such as potassium (K), calcium (Ca), magnesium (Mg), selenium (S), and zinc (Zn) are found in chicory seeds, when compared with those of alfalfa seeds [42]. Elsewhere, [40] identified phosphorus, potassium, calcium, magnesium, sodium (mg g^−1^), iron (mg g^−1^), copper (*μ*g g^−1^), zinc (*μ*g g^−1^), and manganese (*μ*g g^−1^) as the minerals present in chicory seeds. Some researchers [43] isolated cichotyboside, a sesquiterpene glycoside, from* Cichorium intybus *seeds, which was verified to exhibit a significant hepatoprotective activity in rats against carbon tetrachloride induced hepatic damage. Chicory seed can therefore be said to be a healthy alternative and/or complementary nutritional component in animal and human diets.

In 2002, Nørbæk and coworkers [44] identified anthocyanins as the contributory factor for the blue colour of the perianth in chicory flower and [45, 46] later reported that chicory flower contains saccharides, flavonoids, cichorine, methoxycoumarin, and essential oils. Earlier, these scientists [2] established that chicory flowers, leaves, and shoots contain inulin, fructose, choline, resin, chicoric acid (dicaffeoyl tartaric acid), esculetin, esculin (esculetin-6-glucoside), cichoriin, umbelliferone, scopoletin, 6,7-dihydrocoumarin, and sesquiterpene lactones and their glycosides. Also, some investigators [47, 48] isolated vitamins A, B_6_, and K from red chicory which contains carotenoids and some minerals as well. In a food chemistry study by [23, 49], chicoric acid was found to be the major compound derived from the methanolic extracts of chicory. Similarly, earlier investigations [45] noted that octane, n-nonadecane, pentadecanone, and hexadecane have been found to be the primary volatile components of chicory. Aliphatic compounds and their derivatives make up the main fraction of the chicory plant, while the minor constituents are mostly terpenoids [50].

## 3. Ethnomedicinal Benefit of Chicory in Livestock Production

Inulin found in chicory is a source of soluble dietary fiber, a prototype prebiotic especially beneficial in monogastric nutrition and also used as a functional food additive [14, 51, 52]. Various studies have reported that prebiotics stimulates the growth of host-beneficial gut bacteria, such as lactobacilli and bifidobacteria for overall beneficial health [53, 54]. In addition, a prebiotic may stimulate the immune system, decrease the levels of pathogenic bacteria in the intestine [55], relieve constipation, decrease the risk of osteoporosis by increasing essential mineral absorption [56] especially calcium, and reduce the risk of atherosclerosis by lowering the synthesis of triglycerides and fatty acids in the liver as well as lowering their serum levels [54]. Interestingly, probiotic inulin has been indicated to reduce boar taint and odorous compounds in colon and rectal contents when included in male pig diets [20, 57, 58].

Some of the plant secondary metabolites (PSM) of the chicory plant have been reported to possess some ethnomedicinal properties. These PSM serve as herbal remedies for ailments and body conditioning in both humans and animals. Saeed et al. (2017) [59] summarized previous opinions on the health benefits of chicory herb, emphasizing its role as a hepatoprotective agent. The nutritional benefits of* Cichorium intybus* in livestock mitigating peroxidation of lipids in serum and organs, as well as improving growth efficiency, reproduction, and health, were explained. Other bioactive properties such as antioxidant, anti-inflammatory, anticancer, antiprotozoal, antidiabetic, antimicrobial, immunological, cardiovascular, hypolipidemic, gastroprotective, analgesic, anthelmintic, productive and reproductive enhancer, and wound healing properties were also described [59]. Volatile oils are found in all parts of the plant but are more concentrated in the roots which have been found to be effective at eliminating intestinal worms [20, 21, 32]. The studies of Hassan et al. (2014) [60] on the effects of different concentrations of chicory* (Cichorium intybus)* and wormwood* (Artemisia absinthium)* extracts against ovine gastrointestinal nematodes showed high effectiveness against ovine gastrointestinal nematode. The concentration of chicory extract (50 mg/ml) and wormwood extract (25 mg/ml) against adult* Haemonchus contortus in vitro* caused death of all the worms after 4 hours [60]. Another study on anthelmintic effect of forage chicory against gastrointestinal nematode parasites established that feeding forage (⩾70% of chicory DM) significantly reduces worm burdens and fecal egg counts of* Ostertagia ostertagi* in experimentally infected calves. Sesquiterpene lactones identified in both the fresh and silage chicory forage were suspected to contribute to the observed anthelmintic effects of dietary chicory [61]. Scientific works by Milala et al. (2009) [62] and other investigators posited that the polyphenolic acid of chicory roots expresses a wide range of health-promoting activities such as anticarcinogenic, anti-inflammatory, antiviral, antibacterial, antimutagenic, antifungal, anthelmintic, immune-stimulating, and antihepatotoxic activity [43, 63, 64] and its antioxidant properties [65, 66]. Likewise, different researchers [63, 67–69] have submitted that chicory PSM can act against the Human Immunodeficiency Virus (HIV), protect the alimentary tract, and influence the reduction of serum cholesterol. Therefore, the supplementary or complementary inclusions of preparations rich in prebiotic saccharides and polyphenols produced from chicory can be used to promote the healthy properties of a diet and, at the same time, act as food and herbal medicament. A study [70] asserted that chicory* (C. intybus)* is an herb that can be used as a source of fibre in pig diets. According to diverse groups of studies [11, 18–21], chicory root is well known for its toxicity to internal parasites. It has since been reported that feeding inulin suppresses parasites such as* Ascaris* [71] and* Trichuris* [72] in pigs. The lowering of the intestinal pH, which is not favorable for the development of the parasite embryo, has been suggested as a possible mechanism. Two different studies [18, 21] established that ingestion of chicory by farm animals resulted in the reduction of worm burdens, which have prompted its widespread use as a feed supplement with a low fiber concentration. The total number of helminths in the abomasum of lambs grazed on chicory was reported to be remarkably reduced in the study conducted by Marley and coworkers, (2003) [73]. Chicory also contains a low quantity of condensed tannins [32, 74–76] and sesquiterpene lactones [77] which may affect protein utilization efficiency in ruminants and can as well reduce intestinal parasites in animals. It is also noted that sesquiterpene lactones in chicory extract inhibited the hatching of sheep's* Haemonchus contortus* eggs [78]. Earlier, some workers [79] reported that a dose-dependent anthelmintic action of extracts rich in condensed tannins and sesquiterpenes from* C*.* intybus* were indicated to reduce larval motility of lungworm and gastrointestinal nematodes, using a larval migration and inhibition assay. A study on pigs in Denmark [80] has shown chicory to have a positive effect on parasites and* Lawsonia *bacteria and it is relatively inexpensive but when used too much, it can cause a feeling of congestion in the digestive tract.

## 4. Chicory: Specialist Livestock Forage

**Figure 3:**
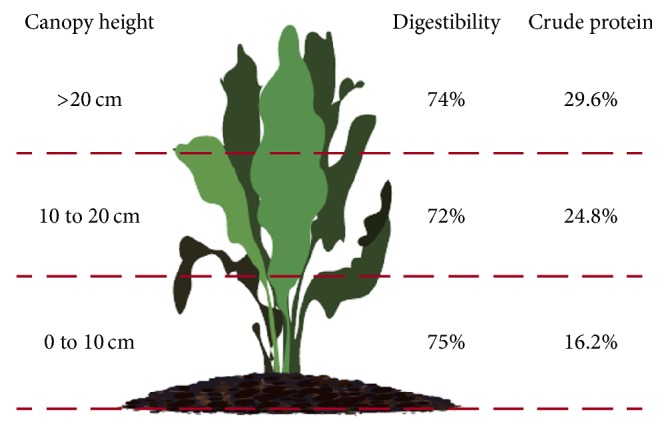
Nutritive values of different parts of a chicory plant [81].

A study [81] revealed that the nutritional value of a chicory crop vary at different parts of the plant, with different growth stages, crop's condition, and the plant's environment as depicted by Figure 3.

Literature has demonstrated that chicory is a suitable forage for lamb finishing. The herb offers exceptional qualities such as thriving well at a minimum annual rainfall of 600 mm and low soil pH levels. The nutritive value of the forage is appreciably high: 14–24% CP, 70–80% digestibility in the leaves, and ME of 13.7 MJ/kg DM [82]. Chicory provides condensed tannins and other secondary metabolites, which positively affect the internal parasites in lambs, lowers the amount of methane production, and increase the reproductive rate in sheep. It is noted in a comparative study that faster growth rate is observed in lambs grazing on chicory (190–370 g/day) than those grazing on other pastures (e.g., ryegrass at 160–230 g/day or lucerne at 170–300 g/day) [83]. Hopkins et al. (1995) [84] also recorded that lambs that grazed chicory on their finishing phase showed no change in meat quality and were not fatter than the lambs fed lucerne forage.

In 2004, Brown and Moot's study [85] established that combined herbage quality and greater herbage consumptions of lucerne forage afforded 30% greater annual crude protein (CP) and ME intake for sward than chicory or red clover. This indicates that lucerne has greater potential to improve livestock production. The report also revealed that chicory, lucerne, and red clover gave similar high ME (10.9–11.3 MJ/kg DM) contents. Lucerne and red clover gave high CP contents of 0.25–0.29 g/g DM, while chicory had a lower CP content of 0.18 g/g DM. However, another study [86] pointed out that the chicory's greater efficiency of use in the rumen can compensate for its lower CP content influence.

In New Zealand, chicory stays leafy in the first summer but in a second summer it goes to seed, which reduces the feed quality [29]. A comparative study between chicory and ryegrass established that herbage quality of chicory of 0–20% stem has a lower DM content than ryegrass pasture (Table 4). Also, chemical constituents of chicory comprises less fibre, more protein, more soluble sugars, and greater mineral content (P, K, S, Ca, Mg, Na, Zn, Cu, and B) than the ryegrass pasture. Though chicory's high digestibility and low fibre content are not suitable as a sole diet for cows, its high digestibility permits it to be more quickly removed from the cow than perennial ryegrass, providing an opportunity to increase voluntary feed intake [29]. The use of chicory in summer in New Zealand varies with pasture availability and quality. A drop in pasture quality in summer warrants the inclusion of chicory, which relatively increases milk solids production per cow compared to a pasture diet [29]. Though chicory can generate high nitrate levels, which rarely pose toxicity issues (probably because chicory is a small portion of the diet), it is still used seasonally to boost pasture quality and quantity in some countries.

A recent review of the problems of feeding dairy cows in Australia and New Zealand indicated that dairy farms that rely on pastures are usually challenged with feed shortages in summer-autumn seasons because of soil moisture deficits [87]. As a result, farmers would require supplementary feed to maintain milk production. Literature has shown that, in summer, chicory and plantain afford more DM and better nutritive qualities than perennial grasses [74]. Another report [17] indicated that chicory and plantain mixture also increases milk production in livestock.

## 5. Antinutritional Properties of* Cichorium intybus*

Some researchers have reported the possibilities of some PSM having detrimental or toxic effects on the growth and performance of livestock once they have exceeded an optimal intake level [88]. Alkaloids, terpenes, saponins, lactones, glycosides, and phenolic compounds are classes of active PSM whose excessive consumption can harmfully affect the health of both ruminants and nonruminants and, in extreme cases, can threaten their survival [21]. For example, the consumption of tannins and other PSM at high intake rates have been associated with reduced feed intake and dry matter digestibility and impaired rumen metabolism when included in the diets of ruminants at more than 4-5% of dry matter [89]. Weight loss, toxicity, and death of animals have also been reported [90, 91]. Earlier studies [21, 88, 90, 92–94] have proved that saponins and condensed tannins are also responsible for mucosal toxicity, reduction in nutrient absorption, and, subsequently, growth impairment. They also recounted that these PSM have been associated with haemolytic action and bloat in ruminants as well. Prior to this, it was purported that excessive consumption of alkaloids, glycosides, and terpenoids by animals can result in lesions in the nervous system [95].

Similarly, monogastrics are not immune to the detrimental fallouts of these phytochemicals. In poultry, tannin levels from 0.5–2.0% can suppress growth and egg production. Between 3 and 7% inclusion levels of tannins in their diet may be lethal [93, 96, 97]. Similar harmful effects of condensed tannins and other plant chemicals have also been reported in swine [93]. However, despite their antinutritional properties, low levels of PSM have been noted to increase growth and milk yield in sheep and cattle [61, 92–94].

## 6. Conclusion

*Cichorium intybus* is grown and used in many parts of the world for various purposes. It is often used for its therapeutic and prophylactic quality, or for maintaining general wellbeing. As a very versatile plant, it is beneficial to both animals and humans due to its high amounts of proteins, carbohydrates, minerals, and phytobioactive elements. In livestock production, it has been noted that some of its phytoconstituents possess properties that improve the welfare of animals either in a parasitized state or otherwise. This makes chicory an ideal, cheap, natural, and sustainable livestock supplement or alternative feed material. However, caution should be exercised when chicory is included in diets or grazed by ruminants to prevent toxicity in high concentrations of PSM. Further research on the multipurpose properties of the phytobioactive elements found in chicory, their antinutritional effects, effective dose of inclusion in animal diets, mechanism of action involved, and the biochemical description of the active PSM is strongly recommended.

## Figures and Tables

**Table 1:** Macronutrient composition of chicory plant^*∗*^ [1].

Chemical composition%	Roots	Leaves
Moisture content	75.63 ± 0.39	83.06 ± 1.55
Crude protein	4.65 ± 0.25	14.70 ± 1.03
Crude ether extract	1.69 ± 0.71	3.68 ± 0.19
Ash	4.25 ± 0.11	10.91 ± 1.86
Total carbohydrates	89.41 ± 1.07	70.71 ± 3.08
Total soluble sugars	11.06 ± 1.00	7.80 ± 1.45
Inulin	44.69 ± 0.88	10.95 ± 2.56
Crude fiber	5.12 ± 1.55	16.78 ± 2.20
Dietary fiber (DF)		
Insoluble DF	30.73 ± 0.33	ND^*∗∗*^
Soluble DF	0.42 ± 0.07	ND^*∗∗*^
Total DF	31.15	ND^*∗∗*^

^*∗*^On dry weight basis; mean ± 5.0 (each value represents the average of three determinations ± standard deviation); ^*∗∗*^ND: not determined.

**Table 2:** Mineral content (mg/100 g) of chicory plant (leaves and roots) in comparison with RDA^*∗*^ [1].

Chicory plant	Macroelements					Microelements			
	Ca	K	Mg	Na	Fe	Cu	Mn	Zn	Pb
Roots	181.26 ± 4.40	103.7 ± 4.62	20.14 ± 1.69	67.42 ± 2.45	1.77 ± 0.21	0.36 ± 0.02	0.31 ± 0.10	0.39 ± 0.03	0.04 ± 0.003
Leaves	292.61 ± 13.35	166.57 ± 3.43	6.944 ± 5.86	88.84 ± 2.58	9.178 ± 0.85	0.60 ± 0.06	0.90 ± 0.01	0.91 ± 0.03	0.03 ± 0.01
RDA mg/100 g	1000–1300	-	240–420	1600	8–11	0.8–1.2	1.6–2.3	12–15	-

^*∗*^RDA recommended daily requirement for men and women.

**Table 3:** Chicory extracts (%) as identified by HPLC [1].

Chicory	Methanolic extracts (%)	Total phenolic content^*∗*^	Phenolic compound (%)	
Leaves	23.16	26.4 ± 1.05	Gallic acid	1.96

Roots	10.75	20.0 ± 0.9	Protocatechuic acid	2.50
			Chlorogenic acid	17.84
			p-Hydroxybenzoic acid	11.04
			Caffeic acid	35.22
			Isovanillic acid	1.97
			p-Coumaric acid	9.65
			Unknown compound	19.46
			Protocatechuic acid	1.77
			Chlorogenic acid	10.85
			Caffeic acid	24.36
			m-Coumaric acid	27.90
			p-Coumaric acid	25.03
			Unknown compound	10.09

^*∗*^Values expressed as mg GAE g^−1^ dry extract (mean of three replicates ± standard deviation).

**Table 4:** Herbage quality of chicory and pasture [29].

	DM (%)	Protein (% DM)	Soluble sugars + starch (% DM)	Fibre (% DM)	Digestibility (% DM)	ME (MJ/kg DM)
Chicory	7–15	16–27	10–22	20–30	72–83	11.5–13.0
Ryegrass^*∗*^	10–30	12–28	8–21	40–55	65–85	9.5–12.5

DM = dry matter; ME = metabolisable energy. Quality may be outside these ranges depending on pasture/crop management. ⁡⁡^*∗*^Spring to autumn.
